# The Effect of Oral Administration of dsRNA on Viral Replication and Mortality in *Bombus terrestris*

**DOI:** 10.3390/v7062765

**Published:** 2015-06-18

**Authors:** Niels Piot, Simon Snoeck, Maarten Vanlede, Guy Smagghe, Ivan Meeus

**Affiliations:** Laboratory of Agrozoology, Department of Crop Protection, Faculty of Bioscience Engineering, Ghent University, 9000 Ghent, Belgium; E-Mails: simonp.snoeck@ugent.be (S.S.); maarten.vanlede@gmail.com (M.V.); guy.smagghe@ugent.be (G.S.); ivan.meeus@ugent.be (I.M.)

**Keywords:** IAPV, dsRNA, bumblebee, virus, immune response

## Abstract

Israeli acute paralysis virus (IAPV), a single-stranded RNA virus, has a worldwide distribution and affects honeybees as well as other important pollinators. IAPV infection in honeybees has been successfully repressed by exploiting the RNA interference (RNAi) pathway of the insect’s innate immune response with virus-specific double stranded RNA (dsRNA). Here we investigated the effect of IAPV infection in the bumblebee *Bombus terrestris* and its tissue tropism. *B. terrestris* is a common pollinator of wild flowers in Europe and is used for biological pollination in agriculture. Infection experiments demonstrated a similar pathology and tissue tropism in bumblebees as reported for honeybees. The effect of oral administration of virus-specific dsRNA was examined and resulted in an effective silencing of the virus, irrespective of the length. Interestingly, we observed that non-specific dsRNA was also efficient against IAPV. However further study is needed to clarify the precise mechanism behind this effect. Finally we believe that our data are indicative of the possibility to use dsRNA for a broad range viral protection in bumblebees.

## 1. Introduction

Managed bees are used worldwide as pollinators to aid food production in open field as well as in greenhouses [1]. Honeybees and bumblebees are the two most commonly used species for commercial pollination purposes. Domesticated honeybees are hosts to multiple viruses [2,3,4], but also different bumblebee species have been reported to carry viruses [5,6]. Managed bees could act as reservoirs for pathogens, and spillover of pathogens from managed bees toward wild pollinators could disrupt natural host-pathogen interactions [7]. Mainly for protozoan parasites from reared bumblebees this phenomena has been studied [8,9,10,11,12], but also disturbance of host-pathogen interaction in wild bumblebees by domesticated honeybees could be happening [6,13]. The risks associated with pathogen spillover could in certain geographic regions be one of the drivers responsible for the decline of pollinators [7,14]. Pathogen eradication in managed bees can already prevent initial spillover events. For honeybees the administration of dsRNA has been successfully applied to lower infection levels of several pathogens, for example Israeli acute paralysis virus (IAPV), Deformed wing virus (DWV), Chinese sacbrood virus (CSBV) and *Nosema ceranae* [15,16,17,18,19]. For bumblebees oral administration of dsRNA has remained unexplored, although genome analysis revealed the presence of the RNAi pathway [20] responsible for processing dsRNA into short interfering RNA (siRNA). These siRNAs target RNA with the corresponding sequence which leads to cleavage of the targeted RNA with the help of Dicer-2. Indeed injection of gene-specific dsRNA can induce gene silencing in bumblebees [21] and also IAPV-specific silencing was obtained after injection of virus-specific dsRNA [22]. This RNAi mechanism has long been reasoned to be the main antiviral immune response in invertebrates as they would lack a non-specific antiviral response. However more recent reports also suggest the presence of a non-specific antiviral response in invertebrates triggered by nucleic acids, as dsRNA, that are recognized as pathogen-associated molecular patterns (PAMPs) [23,24,25]. Still, more research is needed to clarify the precise pathways of this response.

In contrast to honeybees, bumblebees are reared in a closed environment, therefore it should be feasible to produce pathogen-free bumblebees via a combination of screening and preventative measures [26]. dsRNA could help to achieve this goal. Although caution is needed, as in honeybees treatment with dsRNA cannot eliminate the infections [15,17,18], it can significantly lower the viral titer, and thereby perhaps reduce it to titers below the threshold for horizontal transmission. Another option is the application of dsRNA as a preventive treatment. Here we hypothesize that treatment with dsRNA, prior to infection, could prime the bumblebee, making virus infection less likely. This priming could not only eliminate viral spread within breeding facilities, but could also immunize bumblebees when they are used in the field. Indeed aside from the above discussed pathogen spillover mechanism, spillback principles are often overlooked [27]. In spillback the introduced species, in our case a managed bumblebee, could be picking up local parasites or viruses and act as a new host disturbing natural host-pathogen interactions. Here we reported on how different dsRNA treatments can lower infection with IAPV, a virus present in honeybees and bumblebees, causing damage in both species and possibly other pollinators.

## 2. Materials and Methods

### 2.1. Viral Stock

For the production of the viral stock we obtained 240 adult bumblebees from virus-free colonies (Biobest, Westerlo, Belgium). One hundred twenty bumblebees were injected with 2 µL of IAPV solution containing 200 virus particles. The starting virus solution originated from honeybees and was kindly provided by Joachim de Miranda (Swedish University of Agricultural Sciences, Uppsala, Sweden), and quantified by transmission electron microscopy (CODA-CERVA, Brussels, Belgium) as described by Meeus *et al.* [28]. The other 120 bumblebees were injected with nuclease free water and served as a control. After injection bumblebees were kept in micro-colonies of 10 workers at 30 °C and 60% RH (MLR-352 incubator, Sanyo/Panasonic, Osaka, Japan) and *ad libitum* access to 50% sugar water (Biogluc^®^, Biobest) [29]. Four days after injection the bumblebees were stored at −80 °C until the virus purification. Bumblebees were crushed in 0.01 M phosphate buffer (pH 7.0) 0.02% diethyl dithiocarbamate. The exoskeletons were discarded and the remaining liquid was centrifuged (20 min 800× *g*). Supernatant was collected and centrifuged at 40,000× *g* for 4 h at 4 °C. The pellet was suspended in nuclease free water and stored at −80 °C. We performed a quality control on both the viral stock and the control inoculum. Only the virus stock contained IAPV, checked with primers described by Cox-Foster *et al.* [30]. The virus titer was checked with transmission electron microscopy (CODA-CERVA) resulting in 1 × 10^8^ virus particles/µL. We screened for possible contaminating viruses in both viral stock and control inoculum, we tested for slow bee paralysis virus, Kashmir bee virus, acute bee paralysis virus and DWV with PCR and all were negative. Finally, the infectivity of the prepared viral stock and control inoculum solution was tested by injection of 2 µL control inoculum and viral stock. Four days after injection the infection status was determined with PCR [30]. The viral stock solution could successfully infect, and no infection was detected when 2 µL of the control inoculum was injected.

### 2.2. IAPV Infectivity and Mortality

Bumblebees of the same age were kept in micro-colonies per treatment, in an incubator (MLR-352, Sanyo/Panasonic) at 30 °C, 60% RH and constant darkness. Bees had *ad libitum* access to 50% sugar water (Biogluc^®^, Biobest, Westerlo, Belgium) [29]. Prior to feeding bumblebees were starved for 4 h. Bumblebees were inoculated individually in separate boxes (Ø 9 cm) with water (*n =* 17), or with 1 × 10^7^ (*n =* 16), 2 × 10^7^ (*n =* 16) and 1 × 10^8^ (*n =* 18) virus particles in a 20 µL droplet of 50% sugar water (Biogluc^®^, Biobest). Feeding was done under red light, to minimize disturbance of the bees. After inoculation behavior and mortality were followed for 20 days. After 20 days, the infection status of the bees was checked by PCR as described by Cox-Foster *et al.* [30]. Survival analysis was done in SPSS Statistics (version 22.0, IBM Corp, Armonk, NY, USA) with the Mantel-Cox (log rank) test. At day 4 and day 7 post infection 2 bumblebees fed with either 1 × 10^7^ or 1 × 10^8^ were sacrificed for tissue tropism analysis, these bumblebees were not included in the survival analysis.

### 2.3. dsRNA Administration and Effects on Infectivity and Mortality of IAPV Infected Bees

To test the effect of dsRNA on IAPV infectivity and mortality, we fed dsRNA on a daily basis for 6 consecutive days to bumblebees of the same age. Each day a 4h-starved bee received 2 µg of dsRNA in 20 µL of 50% sugar water (Biogluc^®^, Biobest). On the third day the bees also received 1 × 10^7^ virus particles suspended in 20 µL of 50% sugar water (Biogluc^®^, Biobest) [29]. Bees were fed individually as described earlier. In the first experiment bees were put in micro-colonies per treatment after each feeding session and behavior and mortality were scored for 22 days. In the second experiment bees were kept individually during the whole course of the experiment. Five days after the last dsRNA feeding day bumblebees were sacrificed and stored at −80 °C until extraction to determine the viral titer.

### 2.4. Extraction and Virus Quantification

The gut, fat body and head of the bumblebees was dissected and crushed in 350 µL RLT buffer (supplied with the Qiagen RNeasy Mini kit). Further RNA extraction proceeded according manufacturer’s protocol (RNeasy Mini Kit, Qiagen, Venlo, The Netherlands) and were stored at −80 °C until further use. RNA concentrations and purity were determined using a NanoDrop ND-1000 spectrophotometer. A two-step real time RT-PCR was used for quantification of IAPV. First cDNA was made (SuperScript^®^ II Reverse Transcriptase, Life Technologies, Carlsbad, CA, USA) using 500 ng of RNA and oligo-dT primers, according to manufacturer’s protocol. The cDNA was 10 times diluted and IAPV was quantified using a CFX96™ Real-Time PCR Detection system (Bio-Rad, Hercules, CA, USA), performing each reaction in duplicate. The total reaction volume of 20 µL contained 10 µL GoTaq^®^ qPCR Master Mix, (Promega, Madison, WI, USA), 1 µL (10 µM) Forward and 1 µL (10 µM) Reverse primer (see Table 1) and 8 µL diluted cDNA. Nuclease free water was used as a negative control (NTC). Peptidylprolyl isomerase A (PPIA), reported as a stable reference gene in *B. terrestris* with IAPV infection was used for normalization as described by Niu *et al.* [31]. The qPCR protocol for both IAPV (E = 92.6%) and PPIA (E = 79.3%) reference gene was as follows: 95 °C for 5 min, 40 cycles of 95 °C for 30 s and 60 °C for 1 min. The relative normalized IAPV titer was determined as the IAPV titer after normalization with the reference gene PPIA. All qPCR data was processed with the Bio-Rad CFX Manager 3.0 Software (Bio-Rad).

**Table 1:** Primers used for Israeli acute paralysis virus (IAPV) detection, for virus quantification and for dsRNA synthesis, T7 promotor-sequence not included.

Target	Use	Forward Primer (5′→3′)	Reverse Primer (5′→3′)	Reference
IAPV	PCR	CGAACTTGGTGACTTGAAGG	GCATCAGTCGTCTTCCAGGT	[30]
PPIA	RT-qPCR	TCGTAATGGAGTTGAGGAGTGA	CTTGGCACATGAAGTTTGGAAT	[31]
IAPV	RT-qPCR	CCATGCCTGGCGATTCAC	CTGAATAATACTGTGCGTATC	[31]
dsVP586	dsRNA	ACCTGGAAGACGATTGATGC	CTGCCCACTTCCAAACAACT	This study
dsVP443	dsRNA	TATAGATGCCGCTCCATGTG	CTGCCCACTTCCAAACAACT	This study
dsVP293	dsRNA	ACCTGGAAGACGATTGATGC	GTGGGTTTGACGGGTATCAC	This study

### 2.5. dsRNA Synthesis

The RNA extract from the IAPV stock solution was subjected to a DNase treatment and subsequently to RT-PCR with oligo-dT primers, as described earlier. The fragments corresponding to the structural protein-coding region, dsVP586 (bases 7986-8571; accession: NC_009025.1) were PCR amplified with primers (Table 1) carrying a T7 promotor-sequence at the 5′ end, dsRNA for this target was synthesized for three different lengths, 293 bp (dsVP293); 443 bp (dsVP443); and 586 bp (dsVP586). Primers were designed using Primer3 software [32], dsRNA was further produced according manufacturer’s protocol (MEGAscript^®^ RNA kit, Life Technologies), except in the elution step where nuclease free water was used to elute the dsRNA instead of elution buffer. Eluted dsRNA was subjected to a DNAse treatment. dsRNA for GPF (dsGFP, 455 bp) was produced in the same way, starting from a plasmid containing the nucleotide sequence for GFP. The sequences of all dsRNA fragments were compared to the genome of *B. terrestris* and did not contain any similarity longer than 20 bp [20].

## 3. Results

### 3.1. Infectivity of IAPV in Bumblebees and Its Effect on Survival

Previous studies showed that 0.5 × 10^7^ IAPV particles is near the minimum amount required for oral infection in bumblebees, and reported no significant effect on mortality with this dose [28]. Here we inoculated bumblebee workers with three different doses of IAPV (*i.e.*, 1 × 10^7^, 2 × 10^7^ and 1 × 10^8^) and tested the infection status (*n =* 2, per dose per time point) in different body parts (*i.e.*, head, gut and fat body) after four and seven days. For all treatments we also followed mortality and bee behavior.

**Figure 1:**
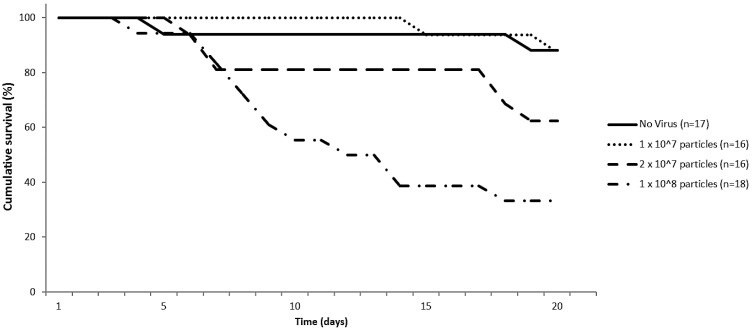
Cumulative survival percentage of bumblebees (*Bombus terrestris*) of the same age fed with different Israeli acute paralysis virus (IAPV) concentrations.

After a single oral administration of 1 × 10^8^ virus particles, we were able to detect IAPV in the head, gut and fat body both four and seven days post infection (p.i.). Feeding of 1 × 10^7^ virus particles had a slower infection progression as IAPV was only detectable in the gut at 4 days p.i., while it took until day seven for virus to appear in the other tested body parts. This reduced infection progression was also apparent in the survival scoring of the bumblebees. Figure 1 shows that feeding of 1 × 10^8^ IAPV virus particles resulted in significant increased mortality after 20 days (*p* = 0.001, Mantel-Cox) compared to the no virus control. Feeding of 2 × 10^7^ virus particles resulted in reduced survival, although not significant (*p* = 0.09, Mantel-Cox). When 1 × 10^7^ virus particles were fed, there was nearly no increase in mortality, which is consistent with previous reports showing only sub-lethal effects [28]. IAPV therefore seems to have a similar pathology in bumblebees as in honeybees. Indeed high mortality within 10 days after feeding of IAPV was reported for honeybees [33]. Experiments comparing doses in honeybees and bumblebees and the naturally occurring titers of IAPV are needed to draw further conclusions.

Looking at the behavior of the bumblebees we noticed disorientated movements: the bumblebees moved around lifting their paws high in the air and tipping over frequently. These observations were not recorded before, and were only seen when high amounts (1 × 10^8^ or 2 × 10^7^) of virus particles were fed and shortly before dying. In honeybees infected with IAPV similar symptoms of spasms have been reported shortly before dying [33]. Darkening of the abdomen is described for IAPV infected honeybees as an early symptom [33], while in bumblebees no such symptoms were recorded in our experiments.

Although there is no knowledge on IAPV titers in naturally infected wild bumblebees, titers of Acute bee paralysis virus, a close related virus have been found to range from 10^4^ to 10^11^ genome copies per bumblebee [6].

### 3.2. Effect of Oral Application of dsRNA on IAPV Infectivity, Relative Virus Titer and Bumblebee Survival

The aim of this experiment was to evaluate if oral administration of dsRNA specific for IAPV could prevent infection in bumblebees. We opted to administer 1 × 10^7^ virus particles to measure the effect of dsRNA on the infection success. Administration of dsVP586 (*n =* 10) and dsGFP (*n =* 10) three days before and three days after inoculation could not prevent infection. After eight days all tested body parts (*i.e.*, head, gut and fat body) (*n =* 5, each treatment) were infected.

**Figure 2:**
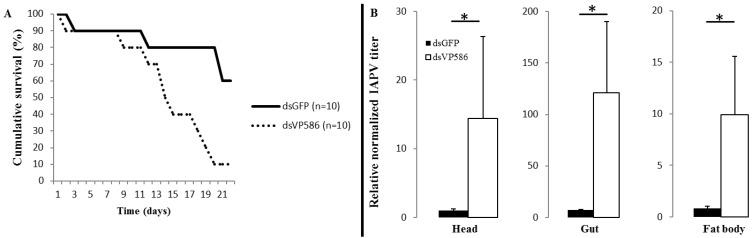
(**A**) Cumulative survival percentage of bumblebees (*Bombus terrestris*) infected with 1 × 10^7^ IAPV particles and fed with virus-specific dsVP586 (*n =* 10), and non-specific dsGFP (*n =* 10); (**B**) Relative normalized IAPV titer in the head, gut and fat body of bumblebees fed with the virus-specific dsVP586 (*n =* 5) and the non-specific dsGFP (*n =* 5) relative to the dsGFP treatment in the head; asterisks indicate significant differences (*p* < 0.05).

In Figure 2b, the relative IAPV titers of both the dsVP586 and dsGFP treatment are compared at eight days p.i. in different body parts. We detected a markedly lower relative normalized virus titer in the head (*p* < 0.001), the gut (*p* = 0.015) and the fat body (*p* = 0.014) in the dsGFP-treated samples compared to the dsVP586 treatment. This result was somewhat unexpected, but actually the impact of non-specific dsRNA (*i.e.*, dsGFP) and the IAPV-specific dsRNA (*i.e.*, dsVP586) on mortality followed the same pattern. The dsGFP control had a significantly higher survival compared to the virus-specific treatment (Mantel-Cox, *p* = 0.009) (Figure 2a). The dsGFP control showed a survival of 60% after 22 days compared to only 10% survival for the dsVP586 treatment. Due to the design of the experiment, where we wanted to test if virus-specific dsRNA could prevent infection, dsGFP served as a control and no virus-only control was used, as infection success with no treatment was proven in the first experiment. Therefore we cannot draw conclusions on the silencing efficiency, however the observed effect of dsGFP compared to the virus-specific dsRNA treatment remains noteworthy. This effect contradicts with previous studies on the use of dsRNA on IAPV infected honeybees, where the non-specific dsRNA control, also dsGFP, had no effect on the survival compared to the virus-specific dsRNA [15]. For DWV infected adult honeybees a slight positive effect of dsGFP on the survival was reported, although the specific dsRNA was significantly better than the dsGFP treatment [18]. Feeding of dsRNA specific for CSBV could prevent infection in larvae as opposed to the unrelated dsGFP, for which there was also no effect on survival [17].

Additionally, the effect of dsGFP in bumblebees on the relative viral titer seems much higher than reported in honeybees. Maori *et al.* [15] reported no effect of dsGFP on the relative viral titer of IAPV infected honeybees in contrast to a significant reduction after virus-specific dsRNA was administered. Nevertheless the positive effect of administering non-specific dsRNAs on virus titers has been reported in invertebrates. A study, although working with an artificial virus (GFP recombinant Sindbis virus), reported a positive effect of non-specific dsRNA on the viral titer in honeybees [24]. In shrimp similar results have been reported where non-specific dsRNAs had a positive effect on survival after infection with Taura syndrome virus (TSV) and White spot syndrome virus (WSSV) [25].

### 3.3. Confirmation of Lower Relative Virus Titers after Non-Specific and Specific dsRNA Treatment

**Figure 3:**
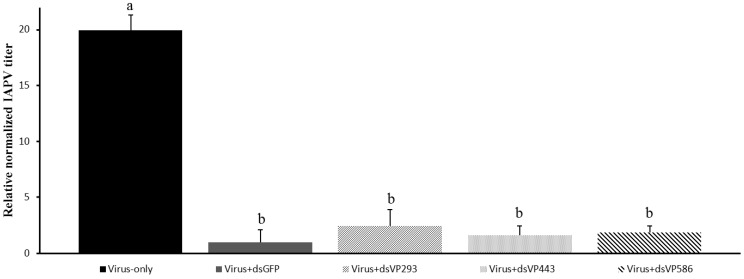
Relative normalized IAPV titer in the head of bumblebees (*Bombus terrestris*) of the same age fed with: only virus; unrelated dsRNA (dsGFP); virus-specific dsRNA of three different lengths, dsVP293, dsVP443 and dsVP586; *n =* 5 for each treatment; relative to the dsGFP treatment in the head. Different letters indicate significant difference (*p* < 0.05).

The lower effect observed for the virus-specific dsRNA (dsVP586) could be due to the differences in length of the dsRNA. The used dsVP586 is much longer than the dsGFP fragment. Indeed size can be a limiting factor in uptake of the dsRNA molecules [34]. To elucidate if the observed effect was due to the length of the dsRNA molecules, the same experiment was repeated. In this second experiment dsRNA molecules specific for IAPV were synthesized in three different lengths, all targeting the same region of the IAPV genome: dsVP293 shorter than dsGFP, dsVP443 with approximately the same length, and dsVP586 the same molecule used in the first experiment. A virus-only control was also used to assess the silencing efficiency of the different treatments. Here only the head was used to compare the impact of the dsRNA on the virus titers, as extraction of the head resulted in less variation in quantity and quality of the RNA extracts; in less variation between biological replicates; and in higher significant effects in the first experiment. Figure 3 gives the relative virus titer in the head of a virus-only control (oral inoculation of IAPV and later on sugar water instead of dsRNA), dsGFP administration as well as all three different IAPV-specific dsRNA treatments. Here all virus-specific treatments showed a significant drop in virus titers compared to the virus-only control (*p* < 0.05). The length of the dsRNA molecule did not have a significant effect on the virus silencing with the IAPV-specific fragment, as there was no significant difference in virus titer between all three virus-specific fragments (*p* > 0.10). The viral titer of the dsGFP control was significantly different from the only-virus control (*p* < 0.05) but not from the virus-specific treatments (*p* > 0.10). These findings confirm the positive effect of dsGFP found in the first experiment.

## 4. Discussion

### 4.1. Pathology and Tissue Tropism of IAPV in Bumblebees

IAPV and other dicistroviruses have been reported in several key pollinators as well as in other insects belonging to different orders [35]. Active replication of IAPV has also been demonstrated in the bumblebee *Bombus impatiens* by detection of the negative strand [36]. However there is still a lack of information concerning the pathology of these dicistroviruses in wild non-*Apis* pollinators [35]. As these viruses have been shown to be able to transmit between honeybees and bumblebees [5], the impact of infections needs to be addressed for these wild non-*Apis* pollinators. We reported here on the pathology and tissue distribution of IAPV infection of *B. terrestris*. Infection with IAPV seems to have similar effects on mortality as reported for honeybees when high doses are fed [15,33]. The tissue tropism also seems similar, where in honeybees IAPV can also be found in the head, the gut and the fat body [37]. At colony level there is however no information on the impact of IAPV in bumblebees, except for artificial micro-colonies consisting of only workers [28]. This is the next step that needs to be taken, as we provide the first evidence of the impact of IAPV on single workers, the impact on queens and colony development is still missing. Indeed wild nest are very hard to find, but studying the impact on colony level is also of importance as wild bees often go through more harsh conditions compared to honeybees [38]. The biological relevance of feeding such high doses of virus particles to look at pathology remains to be tested for IAPV. On the other hand, viral loads as high as 1 × 10^9^ RNA copies have been detected in bee feces for chronic bee paralysis virus (CBPV) [39], giving some idea about biological relevant doses that can be a source for infection. IAPV has also been detected in honeybee feces although there are no quantitative data so far [37]. Indeed fecal-oral transmission is suggested as one of the main transmission routes between taxa [9,40]. This can happen through shared flower use where feces could contaminate the exterior of the bee and thereafter the nectar as well as the pollen of flowers [5]. The impact of IAPV on wild bees needs further investigation as we provide initial evidence that this virus may also be detrimental to bumblebees as is reported for honeybees [41,42].

### 4.2. dsRNA As Possible Treatment to Prevent Spillback Principles

The risk of pathogen spillover associated with the use of commercial bumblebees is well described [8,40,43,44]. However, the use of pathogen free commercial bees can also disturb the natural host-pathogen equilibrium, as these bees can be competent hosts for the native pathogens and so increase the pathogen prevalence, the so called spillback mechanism [27,45]. Here we investigated if oral administration of dsRNA could be of help to prevent IAPV infection in bumblebees, this in an effort to prevent spillback of viruses in pathogen free managed bees. In honeybees this technique successfully lowered the viral titer and prolonged the lifespan of infected bees [15,16,17,18]. Administration of dsRNA was not able to prevent infection with IAPV in bumblebees. Feeding of dsRNA could significantly prolong the lifespan of the infected bumblebees and significantly lower the virus titer. It therefore seems that initial infection of naturally occurring viruses will not be prevented, but the reduced viral replication holds promising results. Indeed dsRNA treatment of managed bees could in this way prevent massive infection loads of these bees, avoiding spillback of viruses into the environment. If these lower virus titers will indeed diminish the possibility of spreading infectious doses of virus and prevent spillback mechanisms needs to be further investigated.

### 4.3. Does Non-Specific dsRNA Works as A PAMP in Bumblebees?

The effect of IAPV-specific dsRNA molecules on the virus titer suggests the presence of a functional specific antiviral RNAi immune response in bumblebees, as all the genes required for the RNAi machinery are present in the genome of *B. terrestris* [20].

Our results, done in bumblebees, demonstrate that a non-specific dsRNA molecule has the same or even higher potency to silence IAPV. In honeybees, virus-specific dsRNA proofed to be more effective to silence viruses compared to non-specific dsRNA [15,17,18]. This suggests that the role of a virus-unspecific immune response might be of greater importance in bumblebees. It remains speculative if the massive domestication and long term selection towards high honey productive honeybees had its trade-offs toward diminished efficiency of certain innate viral immune pathways. On the other hand no effect of inbreeding was observed for encapsulation and phenoloxidase activity, in honeybees [46]. However, the impact of inbreeding and selection on viral immune responses in honeybees still remains unexplored.

The effect of non-specific dsRNA molecules on virus titer and survival has been reported in several invertebrates including honeybees [23,24], thus a pronounced presence in bumblebee is possible. Actually the amount of evidence supporting the hypothesis that there is also a non-specific antiviral response in invertebrates triggered by nucleic acid molecules, as dsRNA, is rising [23]. Flenniken and Andino [24] persued an attempt to find the immune pathways involved in the virus silencing by non-specific dsRNA. Microarray analysis and qPCR in honeybees after virus infection or administration of non-specific dsRNA, diminishing viral loads, revealed that most up-regulated genes could not be linked with previously reported immune pathways. They suggested the involvement of uncharacterized signal transduction cascades in virus control by non-specific dsRNA [24]. Although it could be that known pathways are still involved but regulation is missed by microarray and qPCR, as these techniques are limited to see regulation on mRNA level. While these techniques miss post-transcriptional regulation like post-translational modification. Furthermore, it could also be that the precise time point of up-regulation of the involved cascade genes was missed, further study is needed to clarify this.

Although the exact immune response pathway is unknown, it seems that non-specific dsRNA acts as a PAMP triggering the anti-viral defense. It could be that PAMP recognition strengthens the conventional RNAi pathway, making it more efficient when the actual virus infects and triggers the virus-specific RNAi machinery. But it could also be that in bumblebees other viral defense pathways like the JAK-STAT, Imd, Toll or Jun-*N*-terminal kinase immune pathways are more or equally important and triggered by non-specific dsRNA molecular patterns.

Feeding experiments in honeybees, all show a clear effect of virus-specific dsRNAs [15,16,18]; injection experiments with Sindbis virus in honeybees on the other hand demonstrated an effect of non-specific dsRNA [24] similar to our results in bumblebees after feeding. We can speculate that the siRNA response in different host and against different viruses is not clear-cut. Aside from the influence of the host and virus on antiviral responses it seems the method of delivery can also have an influence. We hypothesize that the biological importance of a certain immune pathway, be it the siRNA pathway (triggered by virus-specific dsRNA) or other pathways, even for the same virus within the same host can be different, depending on the mode of infection and severity of the infection. Furthermore also the actual fitness of the host, its immunologic competence, and genetic background are at play. This would explain the differences reported between non-specific dsRNA and virus-specific dsRNA in viral silencing efficiency [15,17,18,24,25], as well as the differences observed in our experiments. In both our experiments there was a positive effect of the non-specific dsRNA on the virus titer. In the first experiment this effect was significantly higher compared to the virus-specific dsRNA treatment, however in the second experiment there was no significant difference in the effect between the virus-specific and non-specific dsRNA. As both experiments were conducted at different times, the bumblebees used for the experiments originated from different colonies. During the course of both experiments the environmental conditions were kept constant. However, the genetic and physiological background of the bees could be different. It is known that both food stress [47] and genotype [48,49] can influence the innate immune response of bumblebee workers.

Aside from which pathways is triggered, the fact that non-specific dsRNA can silence IAPV opens avenues to prevent replication of multiple viruses in bumblebees. However, the effect of random dsRNA on other viruses in bumblebees first need to be tested. Although our preventive treatment did not lower the infection success of 1 × 10^7^ virus particles, it could significantly lower the virus titer. This treatment could possibly protect bumblebees from high infections when placed in a natural environment and prevent possible spillback.

## References

[1] Velthuis H.H.W., van Doorn A. (2006). A century of advances in bumblebee domestication and the economic and environmental aspects of its commercialization for pollination. Apidologie.

[2] Chen Y.P., Pettis J.S., Collins A., Feldlaufer M.F. (2006). Prevalence and transmission of honeybee viruses. Appl. Environ. Microbiol..

[3] Ribière M., Ball B.V., Aubert M., Aubert M., Ball B., Fries I., Moritz R., Milani N., Bernardinelli I. (2008). Natural History And Geographical Distribution Of Honey Bee Viruses. Virology and the Honey Bee.

[4] De Miranda J.R., Cordoni G., Budge G. (2010). The Acute bee paralysis virus-Kashmir bee virus-Israeli acute paralysis virus complex. J. Invertebr. Pathol..

[5] Singh R., Levitt A.L., Rajotte E.G., Holmes E.C., Ostiguy N., Vanengelsdorp D., Lipkin W.I., Depamphilis C.W., Toth A.L., Cox-Foster D.L. (2010). RNA viruses in hymenopteran pollinators: Evidence of inter-taxa virus transmission via pollen and potential impact on non-Apis hymenopteran species. PLoS ONE.

[6] McMahon D.P., Fürst M.A., Caspar J., Theodorou P., Brown M.J.F., Paxton R.J. (2015). A sting in the spit: Widespread cross-infection of multiple RNA viruses across wild and managed bees. J. Anim. Ecol..

[7] Meeus I., Brown M.J.F., de Graaf D.C., Smagghe G. (2011). Effects of invasive parasites on bumble bee declines. Conserv. Biol..

[8] Murray T.E., Coffey M.F., Kehoe E., Horgan F.G. (2013). Pathogen prevalence in commercially reared bumble bees and evidence of spillover in conspecific populations. Biol. Conserv..

[9] Graystock P., Goulson D., Hughes W.O.H. (2014). The relationship between managed bees and the prevalence of parasites in bumblebees. Peer J. Prepr..

[10] Schmid-Hempel R., Eckhardt M., Goulson D., Heinzmann D., Lange C., Plischuk S., Escudero L.R., Salathé R., Scriven J.J., Schmid-Hempel P. (2014). The invasion of southern South America by imported bumblebees and associated parasites. J. Anim. Ecol..

[11] Arbetman M.P., Meeus I., Morales C.L., Aizen M.A., Smagghe G. (2012). Alien parasite hitchhikes to Patagonia on invasive bumblebee. Biol. Invasions.

[12] Maharramov J., Meeus I., Maebe K., Arbetman M., Morales C., Graystock P., Hughes W.O.H., Plischuk S., Lange C.E., de Graaf D.C. (2013). Genetic variability of the neogregarine Apicystis bombi, an etiological agent of an emergent bumblebee disease. PLoS ONE.

[13] Fürst M.A, McMahon D.P., Osborne J.L., Paxton R.J., Brown M.J.F. (2014). Disease associations between honeybees and bumblebees as a threat to wild pollinators. Nature.

[14] Goulson D., Nicholls E., Botias C., Rotheray E.L. (2015). Bee declines driven by combined stress from parasites, pesticides, and lack of flowers. Science.

[15] Maori E., Paldi N., Shafir S., Kalev H., Tsur E., Glick E., Sela I. (2009). IAPV, a bee-affecting virus associated with colony collapse disorder can be silenced by dsRNA ingestion. Insect Mol. Biol..

[16] Hunter W., Ellis J., Vanengelsdorp D., Hayes J., Westervelt D., Glick E., Williams M., Sela I., Maori E., Pettis J. (2010). Large-scale field application of RNAi technology reducing Israeli acute paralysis virus disease in honey bees (*Apis mellifera*, hymenoptera: Apidae). PLoS Pathog..

[17] Liu X., Zhang Y., Yan X., Han R. (2010). Prevention of chinese sacbrood virus infection in apis cerana using rna interference. Curr. Microbiol..

[18] Desai S.D., Eu Y.J., Whyard S., Currie R.W. (2012). Reduction in deformed wing virus infection in larval and adult honey bees (*Apis mellifera* L.) by double-stranded RNA ingestion. Insect Mol. Biol..

[19] Paldi N., Glick E., Oliva M., Zilberberg Y., Aubin L., Pettis J., Chen Y., Evans J.D. (2010). Effective gene silencing in a microsporidian parasite associated with honeybee (*Apis mellifera*) colony declines. Appl. Environ. Microbiol..

[20] Sadd B.M., Barribeau S.M., Bloch G., de Graaf D.C., Dearden P., Elsik C.G., Gadau J., Grimmelikhuijzen C.J., Hasselmann M., Lozier J.D. (2015). The genomes of two key bumblebee species with primitive eusocial organization. Genome Biol..

[21] Deshwal S., Mallon E.B. (2013). Antimicrobial peptides play a functional role in bumblebee anti-trypanosome defense. Dev. Comp. Immunol..

[22] Piot N., Meeus I., Smagghe G. Injection of Israeli acute paralysis virus-specific dsRNA reduces virus replication and mortality in *Bombus terrestris*.

[23] Wang P.H., Weng S.P., He J.G. (2014). Nucleic acid-induced antiviral immunity in invertebrates: An evolutionary perspective. Dev. Comp. Immunol..

[24] Flenniken M.L., Andino R. (2013). Non-specific dsRNA-mediated antiviral response in the honey bee. PLoS ONE.

[25] Robalino J., Bartlett T.C., Chapman R.W., Gross P.S., Browdy C.L., Warr G.W. (2007). Double-stranded RNA and antiviral immunity in marine shrimp: Inducible host mechanisms and evidence for the evolution of viral counter-responses. Dev. Comp. Immunol..

[26] Meeus I., Mosallanejad H., Niu J., de Graaf D.C., Wäckers F., Smagghe G. (2014). Gamma irradiation of pollen and eradication of Israeli acute paralysis virus. J. Invertebr. Pathol..

[27] Strauss A., White A., Boots M. (2012). Invading with biological weapons: The importance of disease-mediated invasions. Funct. Ecol..

[28] Meeus I., de Miranda J.R., de Graaf D.C., Wäckers F., Smagghe G. (2014). Effect of oral infection with Kashmir bee virus and Israeli acute paralysis virus on bumblebee (*Bombus terrestris*) reproductive success. J. Invertebr. Pathol..

[29] Mommaerts V., Sterk G., Smagghe G. (2006). Hazards and uptake of chitin synthesis inhibitors in bumblebees Bombus terrestris. Pest Manag. Sci..

[30] Cox-Foster D.L., Conlan S., Holmes E.C., Palacios G., Evans J.D., Moran N.A., Quan P.-L., Briese T., Hornig M., Geiser D.M. (2007). A metagenomic survey of microbes in honey bee colony collapse disorder. Science.

[31] Niu J., Cappelle K., de Miranda J.R., Smagghe G., Meeus I. (2014). Analysis of reference gene stability after Israeli acute paralysis virus infection in bumblebees Bombus terrestris. J. Invertebr. Pathol..

[32] Koressaar T., Remm M. (2007). Enhancements and modifications of primer design program Primer3. Bioinformatics.

[33] Maori E., Lavi S., Mozes-Koch R., Gantman Y., Peretz Y., Edelbaum O., Tanne E., Sela I. (2007). Isolation and characterization of Israeli acute paralysis virus, a dicistrovirus affecting honeybees in Israel: Evidence for diversity due to intra- and inter-species recombination. J. Gen. Virol..

[34] Huvenne H., Smagghe G. (2010). Mechanisms of dsRNA uptake in insects and potential of RNAi for pest control: A review. J. Insect Physiol..

[35] Manley R., Boots M., Wilfert L. (2015). Emerging viral disease risk to pollinating insects: Ecological , evolutionary and anthropogenic factors. J. Appl. Ecol..

[36] Levitt A.L., Singh R., Cox-Foster D.L., Rajotte E., Hoover K., Ostiguy N., Holmes E.C. (2013). Cross-species transmission of honey bee viruses in associated arthropods. Virus Res..

[37] Chen Y.P., Pettis J.S., Corona M., Chen W.P., Li C.J., Spivak M., Visscher P.K., DeGrandi-Hoffman G., Boncristiani H., Zhao Y. (2014). Israeli acute paralysis virus: Epidemiology, pathogenesis and implications for honey bee health. PLoS Pathog..

[38] Goulson D., Lye G.C., Darvill B. (2008). Decline and conservation of bumble bees. Annu. Rev. Entomol..

[39] Ribière M., Lallemand P., Iscache A.L., Schurr F., Celle O., Blanchard P., Olivier V., Faucon J.P. (2007). Spread of infectious chronic bee paralysis virus by honeybee (*Apis mellifera* L.) feces. Appl. Environ. Microbiol..

[40] Otterstatter M.C., Thomson J.D. (2008). Does pathogen spillover from commercially reared bumble bees threaten wild pollinators?. PLoS ONE.

[41] Hou C., Rivkin H., Slabezki Y., Chejanovsky N. (2014). Dynamics of the presence of israeli acute paralysis virus in honey bee colonies with colony collapse disorder. Viruses.

[42] Ribière M., Drajnudel P., Faucon J.-P. (2008). The collapse of bee colonies: The CCD case (“Colony collapse disorder”) and the IAPV virus (Israeli acute paralysis virus). Virology.

[43] Colla S.R., Otterstatter M.C., Gegear R.J., Thomson J.D. (2006). Plight of the bumble bee: Pathogen spillover from commercial to wild populations. Biol. Conserv..

[44] Graystock P., Yates K., Evison S.E.F., Darvill B., Goulson D., Hughes W.O.H. (2013). The Trojan hives: Pollinator pathogens, imported and distributed in bumblebee colonies. J. Appl. Ecol..

[45] Jones C.M., Brown M.J.F. (2014). Parasites and genetic diversity in an invasive bumblebee. J. Anim. Ecol..

[46] Lee G.M., Brown M.J.F., Oldroyd B.P. (2012). Inbred and outbred honey bees (*Apis mellifera*) have similar innate immune responses. Insectes Soc..

[47] Moret Y., Schmid-Hempel P. (2000). Survival for immunity: The price of immune system activation for bumblebee workers. Science.

[48] Schlüns H., Sadd B.M., Schmid-Hempel P., Crozier R.H. (2010). Infection with the trypanosome Crithidia bombi and expression of immune-related genes in the bumblebee Bombus terrestris. Dev. Comp. Immunol..

[49] Wilfert L., Gadau J., Baer B., Schmid-Hempel P. (2007). Natural variation in the genetic architecture of a host-parasite interaction in the bumblebee Bombus terrestris. Mol. Ecol..

